# Psychiatric symptoms and moral injury among US healthcare workers in the COVID-19 era

**DOI:** 10.1186/s12888-021-03565-9

**Published:** 2021-11-05

**Authors:** Doron Amsalem, Amit Lazarov, John C. Markowitz, Aliza Naiman, Thomas E. Smith, Lisa B. Dixon, Yuval Neria

**Affiliations:** 1 New York State Psychiatric Institute and Department of Psychiatry, Columbia University Vagelos College of Physicians & Surgeons, 1051 Riverside Drive, New York, NY 10032 USA; 2 School of Psychological Sciences, Tel Aviv University, Tel Aviv, Israel; 3 New York State Office of Mental Health, New York City, USA; 4 Department of Epidemiology, Columbia University Irving Medical Center, New York, USA

**Keywords:** Healthcare, COVID-19, Depression, Anxiety, PTSD, Moral injury

## Abstract

**Background:**

Emerging cross-sectional data indicate that healthcare workers (HCWs) in the COVID-19 era face particular mental health risks. Moral injury – a betrayal of one’s values and beliefs, is a potential concern for HCWs who witness the devastating impact of acute COVID-19 illness while too often feeling helpless to respond. This study longitudinally examined rates of depression, generalized anxiety disorder (GAD), posttraumatic stress disorder (PTSD), and moral injury among United States HCWs in the COVID-19 era. We anticipated finding high levels of clinical symptoms and moral injury that would remain stable over time. We also expected to find positive correlations between clinical symptoms and moral injury.

**Methods:**

This three-wave study assessed clinical symptoms and moral injury among 350 HCWs at baseline, 30, and 90 days between September and December 2020. Anxiety, depression, PTSD, and moral injury were measured using the Generalized Anxiety Disorder-7 (GAD-7), Patient Health Questionnaire-9 (PHQ-9), Primary Care PTSD Screen (PC-PTSD), and Moral Injury Events Scale (MIES).

**Results:**

Of the 350 HCWs, 72% reported probable anxiety, depression, and/or PTSD disorders at baseline, 62% at day 30, and 64% at day 90. High level of moral injury was associated with a range of psychopathology including suicidal ideation, especially among healthcare workers self-reporting COVID-19 exposure.

**Conclusions:**

Findings demonstrate broad, persisting, and diverse mental health consequences of the COVID-19 pandemic among United States HCWs. This study is the first to longitudinally examine the relationships between moral injury and psychopathology among HCWs, emphasizing the need to increase HCWs’ access to mental healthcare.

## Introduction

The ongoing stress of the COVID-19 pandemic has placed healthcare workers (HCWs) at risk for anxiety, depression, and posttraumatic stress disorders (PTSD) [1]. Healthcare workers already confronted high risks for the negative effects of chronic stress before the pandemic - stress now aggravated by fear, frustration, demoralization, and multiple other challenges [2, 3]. Constantly expected to respond to medical emergencies, HCWs often experience distress about contracting and spreading the disease, with some facing repeated exposure to terminally ill patients who are cut off from family and friends [4]. In addition, they are concerned about staff shortages and competency when redeployed without proper training [4]. Healthcare workers may feel conflicted, caught between their wish to fulfill their duty and their own need to survive the pandemic. In the context of such pressures, studies have shown high psychopathology among HCWs, with recent reviews finding 21–29% self-reported anxiety, 21–26% depression, and 20–29% PTSD [5–9]. Published studies have assessed symptoms cross-sectionally, usually in the early phases of the pandemic or in countries that more quickly contained the spread of the virus (e.g., China). A literature search found no longitudinal studies evaluating mental health trajectories among HCWs during the COVID-19 era. The current study longitudinally assessed the three-month burden of the ongoing COVID-19 pandemic on HCWs in the chronically hard-hit United States (September – December 2020).

At the height of the pandemic, many HCWs had to make crucial life and death decisions about acutely ill COVID-19 patients. These include rationing of care, employing ventilators, and prioritizing who is treated for disease [10]. Consequently, COVID-19 exposed HCWs to experiences that could violate their moral values, potentially causing “moral injury” [11], which is defined as “the lasting stress of perpetrating, failing to prevent, or witnessing acts that transgress or deeply violate one’s moral or ethical code” [12]. An emerging clinical and conceptual literature in veterans and civilians describes moral injury and its detrimental impact on mental health [13]. Moral injuries generate anger, shame, guilt, and mistrust, and have demonstrated associations to functional deficits and psychopathology including PTSD, depression, suicidal ideation, and drug and alcohol abuse [9, 14–16]. Moral injury may be a major concern for HCWs who witness the devastating impact of COVID-19 illness while often feeling helpless to respond adequately. Lack of resources, guidance, and training amplify feelings of incompetence and betrayal by leaders. Although moral injury is not a mental illness per se, it may contribute to the development of mental health problems. To date, however, the general prevalence of moral injury is unknown, and no study has examined moral injury and its association with clinical symptoms among HCWs.

The aftermath of the COVID-19 pandemic holds even graver ramifications for HCWs. While HCWs already face higher suicide risk, rates may further increase during and following the COVID-19 pandemic for several reasons, including concerns of infection, fear of illness, social isolation, traumatic experiences, and increased anxiety and depression [17–19]. Work stress, shortages of staff and necessary personal protective equipment, and overwhelmed facilities are additional pressures. Research found loneliness and reduced access to community support and mental health treatment were associated with suicidal ideation and behaviors [20]. To date, no studies have examined the relationship between COVID-19 and suicidal ideation over time.

In sum, previous studies of the effects of COVID-19 on HCWs were cross-sectional, conducted relatively early in the pandemic, and used a narrow range of measures. We designed this study to longitudinally assess clinical symptoms including suicidal ideation, moral injury, and examine the associations between them. Assessments were conducted at baseline and 30 and 90 days thereafter. We hypothesized that between September and December 2020: 1) HCWs would exhibit high levels of self-reported anxiety, depression, PTSD, and moral injury at baseline and throughout the follow-ups, and 2) clinical symptoms reported by the HCWs would be strongly associated with moral injury.

## Methods

### Participants and recruitment

Amazon Mechanical Turk (MTurk), a leading crowdsourcing tool, has been frequently used in medical and psychology research [21, 22]. Data acquired on MTurk, an easily accessible online platform, have demonstrated validity and reliability across tasks and countries [23]. An emerging trend toward online platforms has been accelerated by the COVID-19 pandemic, altering knowledge dissemination and methods of data collection [24]. To enhance participant verification and quality of data, we recruited participants through *TurkPrime/CloudResearch*, an MTurk-connected internet-based platform specifically designed for data acquisition for social and behavioral research [25]. *TurkPrime* constantly screens the MTurk population to identify appropriately defined participants: ensuring response consistency in demographic and other characteristics (e.g., occupation) over time, blocking participants who use tools to hide their location, running tests to identify VPN usage, and creating unique anonymous IDs for respondents. Between September and December 2020, we recruited English-speaking United States resident HCWs 18–80 years old as part of a larger study examining stigma surrounding treatment and help-seeking intentions [26]. “Healthcare worker” was defined as any individual with a health-related occupation: e.g., nurses, physicians, and emergency medical technicians. Participants were compensated $8 in installments to ensure they completed all time points. The New York State Psychiatric Institute Institutional Review Board approved the study. Participants reviewed an informed consent form before study entry and at every time point.

### Procedure

Participants were directed to complete the study procedures via Qualtrics.com, a secure, online data collection platform. Demographic information, clinical symptoms, and moral injury were first assessed in September 2020. Follow-up assessments were conducted 30 and 90 days following the initial assessment, completing data collection in December 2020.

### Instruments

Clinical assessments included the Generalized Anxiety Disorder-7 (GAD-7), Patient Health Questionnaire-9 (PHQ-9), and the Primary Care PTSD Screen (PC-PTSD). The seven-item GAD-7 assesses the likely presence of generalized anxiety during the past 2 weeks [27] on a 0 (“not at all”) to 3 (“nearly every day”) scale, with an overall score range of 0 to 21. A higher score indicates greater self-reported anxiety. A GAD-7 total score of 5 to 9 indicates mild anxiety, 10 to 14 moderate anxiety, and 15 to 21 severe anxiety. Previous studies demonstrated high sensitivity (89%) and specificity (82%) [27]. In this study, Cronbach’s α was .93.

The nine-item PHQ-9, a screening instrument based on the Diagnostic and Statistical Manual of Mental Disorders, 5th Edition (DSM-5) diagnostic criteria for major depression, assesses depression symptoms during the past two weeks [28]. Response options range from 0 (“not at all”) to 3 (“nearly every day”), with an overall score of 0 to 27. A PHQ-9 total score ranging from 5 to 9 indicates mild depression, 10 to 14 moderate depression, 15 to 19 moderate-to-severe depression, and 20 to 27 severe depression. Previous studies demonstrated high sensitivity of 88% and specificity of 85% [29]. In this study, Cronbach’s α was .90.

The PC-PTSD-5, a five-item yes/no self-report instrument, is designed to identify probable PTSD. It reflects DSM-5 PTSD diagnostic criteria, with three positive items as the screening threshold [30]. The PC-PTSD has good reliability and showed good operating characteristics when compared to PTSD diagnosis based on clinician interviews [31]. To focus the PC-PTSD on COVID-related events, items were adjusted as follows: “In the past month, have you felt guilty or unable to stop blaming yourself or others for COVID-19 related events?” and “Have you felt numb or detached from people, activities, or your surroundings?” In this study, Cronbach’s α was .73.

The 9-item Moral Injury Events Scale (MIES) [32] assessed moral injury. The MIES has good internal consistency and underlying latent factors of perceived transgression by self or others (e.g.,” I saw things that were morally wrong,” and” I acted in ways that violated my own moral code or values”) and perceived betrayals (e.g., “I feel betrayed by leaders I once trusted”). Response choices range from 1 (*disagree*) to 4 (*agree*), with an overall score of 9 to 36. A higher score indicates greater moral injury. For clarity, we collapsed the values into agree (combining “strongly agree” and “agree”) and disagree (“strongly disagree” and “disagree”). In this study, MIES scores showed good internal consistency (α = .87).

### Analysis

Repeated measure ANOVA compared psychopathological characteristics and MIES total scores across time points. Pearson’s correlation coefficient was used to evaluate linear relationships between psychopathology (GAD-7, PHQ-9, PC-PTSD total scores) and MIES total scores across assessments. Spearman correlation coefficient was used to evaluate pairwise associations between psychopathology, including suicidal ideation and COVID-19 exposure, and between suicidal ideation and MIES total scores. COVID-19 exposure was defined as testing positive or having a close friend/family member who received or currently has a COVID-19 diagnosis. A simple linear regression was calculated to predict participants’ level of anxiety (GAD-7), depression (PHQ-9), and PTSD (PC-PTSD) based upon their moral injury total scores (MIES). Preliminary analyses were performed to ensure there was no violation of the assumption of normality, linearity, and multicollinearity. Data were analyzed using SPSS 26.0. A Bonferroni correction for multiple comparisons yielded a corrected *p-value* significance threshold of 0.05/5 = 0.01.

## Results

### Sample demographic characteristics

**Fig. 1:**
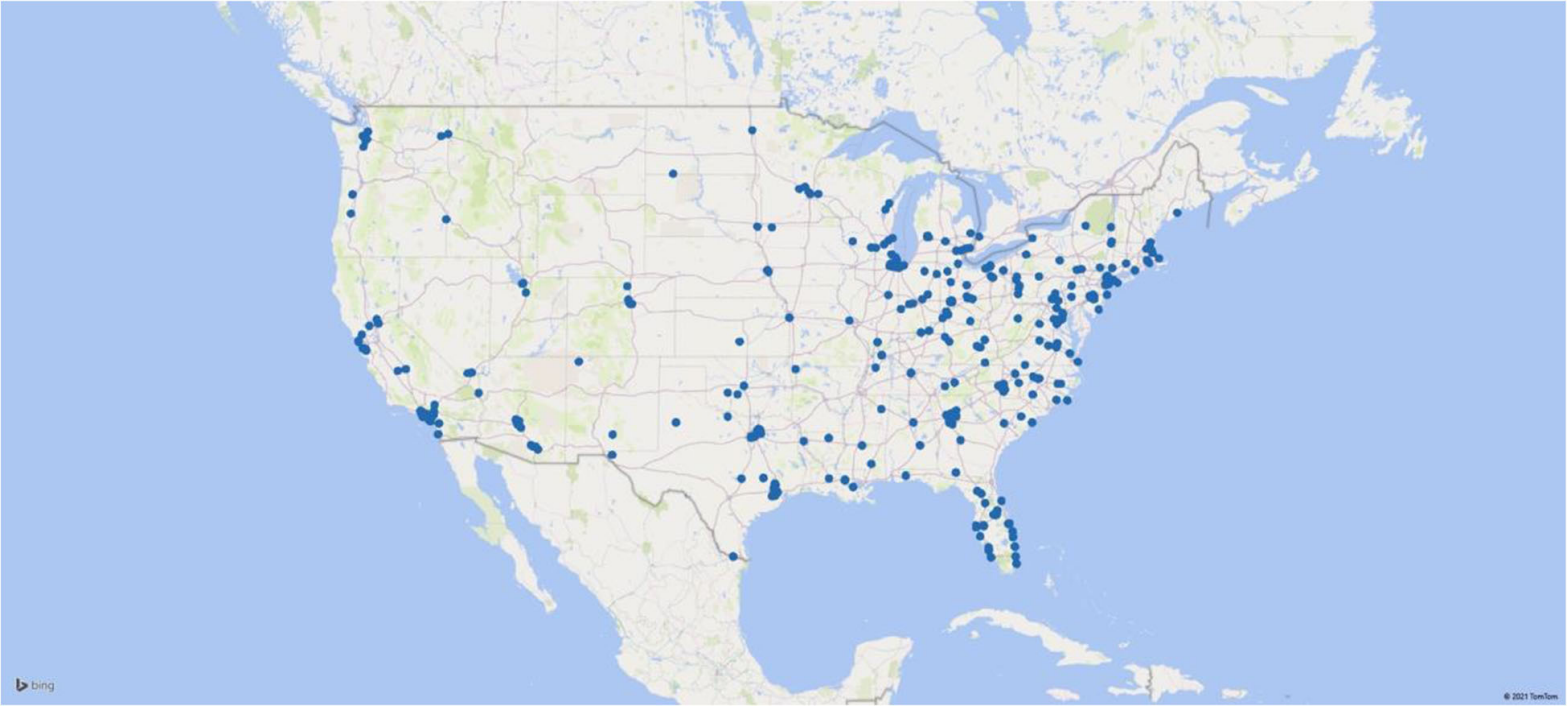
Healthcare worker participant geograohic distribution

A sample of 350 participants completed the first evaluation, excluding 12 (3%) individuals who failed validity tests. Of those 350, 280 (80%) completed the 30-day follow-up, and 267 (76%) completed the 90-day follow-up. Demographic characteristics did not differ across assessments. Mean participant age was 34.8 (SD 11.5; range 18–70). Most respondents were female (*n* = 260, 74%). Seventy-three percent of participants self-identified as white, 13% as African American, 11% as Asian, 1% as Native American, and 1% as other. Ten percent reported Hispanic ethnicity. Participants’ occupations included 68% nurses, 15% physicians, 9% emergency medical technicians, 3% physical therapists, 2% pharmacists, 2% hospital administrators, 1% social workers and other therapists (see Table 1**)** nationwide (Fig. 1). We found no significant differences in clinical symptoms or moral injury across subgroups.

**Table 1:** Demographics, n (%)

Items	*n* = 350
Mean age (SD)	34.8 ± 11.5
Gender – female	260 (74)
Hispanic	34 (10)
Race	
White	256 (73)
African American	46 (13)
Asian	38 (11)
Native American	5 (1)
Other	4 (1)
Occupation	
Nurses	237 (68)
Physicians	52 (15)
Emergency medical technicians	30 (9)
Physical therapists	12 (3)
Pharmacists	8 (2)
Social workers and other therapists	5 (1)
Other	6 (2)

### Sample clinical and moral injury characteristics

Table 2 presents clinical symptoms over time. Of the 350 HCWs, 72% reported probable anxiety, depression, and/or PTSD disorders at baseline, 62% at day 30, and 64% at day 90. Specifically, 215/350 (62%) participants surpassed the generalized anxiety disorder threshold (GAD-7 ≥ 5) at baseline, 146/280 (52%) on day 30, and 144/267 (54%) on day 90 (F = 5.0, *p* = .007). Two hundred-two (58%) participants reached the depression threshold (PHQ-9 ≥ 5) at baseline, 146/280 (52%) on day 30, and 147/267 (55%) on day 90. In response to the PHQ-9 question assessing suicidal ideation (“How often do you have thoughts that you would be better off dead or of hurting yourself in some way?”), 65/350 participants (19%) reported suicidal thoughts; of those, 35 (10% of the entire sample) endorsed “several days,” 19 (5%) “more than half the days,” and 11 (3%) “almost every day.” Rates of PHQ-9 depression and suicidal ideation did not significantly change over time (F = 2.4, *p* = .091, and F = 1.9, *p* = .149, respectively). One hundred twenty-one (35%) respondents reported symptoms suggesting probable PTSD (PC-PTSD≥3) at baseline, which decreased to 78/280 (28%) on day 30 and 64/267 (24%) on day 90 (F = 8.5, *p* < .001). Only 48/350 (20%) respondents never met criteria for probable anxiety, depression, or PTSD.

**Table 2:** Longitudinal Presentation of the Percentage of Positive Cases for Self-Report Anxiety (GAD-7 ≥ 5), Depression (PHQ-9 ≥ 5), Suicidal Ideation (PHQ9, Item 9), and PTSD (PC-PTSD≥3)

Items	Baseline *n* = 350	Day 30 *n* = 280	Day 90 *n* = 267	Statistic^a^	*p-value*
	n (%)	n (%)	n (%)		
*Severity level of anxiety (GAD)*					
Mild	100 (29)	76 (27)	72 (27)		
Moderate	66 (19)	44 (16)	46 (17)		
Severe	49 (14)	26 (9)	27 (10)		
Total	215 (62)	146 (52)	145 (54)	5.0	.007
*Severity level of depression (PHQ)*					
Mild	88 (25)	68 (24)	68 (26)		
Moderate	60 (17)	42 (15)	39 (15)		
Moderately severe	34 (10)	27 (10)	27 (10)		
Severe	20 (6)	9 (3)	12 (5)		
Total	202 (58)	146 (52)	148 (55)	2.4	.091
Suicidal ideation	65 (19)	38 (14)	49 (18)	1.9	.149
PC-PTSD	121 (35)	79 (28)	65 (24)	8.5	.000

^a^ Repeated measure ANOVA; *GAD*-7 Generalized Anxiety Disorder, *PHQ*-9 Patient Health Questionnaire, *PC-PTSD* Primary Care PTSD Screen for DSM-5

Table 3 presents longitudinal scores on the Moral Injury Events Scale (MIES). The agreement percentage of each MIES item and total scores did not differ across assessments, remaining high throughout the 90-day interval (repeated measure ANOVA, F = .18, *p* = .84). For example, roughly half of participants endorsed “I saw things that were morally wrong,” “I am troubled by having witnessed others’ immoral acts,” “I feel betrayed by leaders whom I once trusted,” and “I feel betrayed by others outside the healthcare system whom I once trusted.”

**Table 3:** Longitudinal Follow-Up of Moral Injury Events Scale (MIES) Among Healthcare Workers

Items		Agreement		
		Baseline *n* = 350	Day 30 *n* = 280	Day 90 *n* = 267
		n (%)	n (%)	n (%)
*Transgression by others*				
1	I saw things that were morally wrong	192 (55)	138 (49)	135 (51)
2	I am troubled by having witnessed others’ immoral acts	171 (49)	125 (45)	142 (46)
*Self-transgression*				
3	I acted in ways that violated my own moral code or values	66 (19)	51 (18)	52 (20)
4	I am troubled by having acted in ways that violated my own morals or values	68 (19)	53 (19)	45 (17)
5	I violated my own morals by failing to do something that I felt I should have done	71 (20)	60 (21)	57 (22)
6	I am troubled because I violated my morals by failing to do something that I felt I should have done	72 (21)	56 (20)	46 (17)
*Betrayal*				
7	I feel betrayed by leaders who I once trusted	157 (45)	130 (46)	142 (46)
8	I feel betrayed by coworkers who I once trusted	111 (32)	78 (28)	77 (29)
9	I feel betrayed by others outside the healthcare system who I once trusted	152 (43)	114 (41)	101 (38)
**MIES total scores**		17.8 ± 6.5	17.2 ± 6.8	17.0 ± 6.6

Table 4 presents correlations between a) clinical symptoms (probable anxiety, depression, suicidal ideation, and PTSD), b) MIES, and c) COVID-19 exposure at each time point (baseline, 30, and 90 days). High rates of anxiety, depression, suicidal ideation, and PTSD were associated with high MIES total score across all time points (Pearson Correlation Coefficient ranged from .38 to .59, *p* < .001), and with exposure to COVID-19 only at baseline (Spearman Correlation Coefficient .14 for self-reported depression, *p* < .01, .22 for PTSD, *p* < .001). Regression equations were significant for anxiety (F = 156.4, *p* < .001, *R*^2^ of .31), depression (F = 182.4, *p* < .001, *R*^2^ of .34), and PTSD (F = 75.5, *p* < .001, *R*^2^ of .18). Participants’ predicted GAD-7 total scores increased .49, PHQ-9 total scores increased .57, and PC-PTSD increased .11 for each point of MIES total scores.

**Table 4:** Correlations Between Clinical Symptoms, the Moral Injury Events Scale (MIES), and Exposure to COVID-19 at Baseline, One Month, and Three Months

			Clinical symptoms		
	Day	GAD-7	PHQ-9	Suicidal ideation	PC-PTSD
MIES	1	.56***	.59***	.44***	.42***
	30	.50***	.56***	.39***	.48***
	90	.47***	.47***	.38***	.57***
Exposure to COVID-19^a^	1	.15**	.14**	.001	.22***
	30	.04	.06	.05	.05
	90	.06	.02	.0	.07

*GAD*-7 Generalized Anxiety Disorder, *PHQ*-9 Patient Health Questionnaire, *PC-PTSD* Primary Care PTSD Screen for DSM-5, *MIES* Mural Injury Events Scale, ^an^ Exposure to COVID-19 were participants who tested positive for COVID-19 or had a close friend/family member who tested positive; ***p < .001; **p < .01

## Discussion

Our study assessed healthcare workers’ self-reported levels of anxiety, depression, suicidal ideation, PTSD, and moral injury over 90 days between September and December 2020. Analysis tested associations between clinical symptoms, moral injury, and COVID-19 exposure. As hypothesized, we found high levels of symptomatology and moral injury, both of which remained high across timepoints. Psychopathology and moral injury were correlated across all timepoints. This is the first study to demonstrate such associations and longitudinal trajectory of the mental health consequences of the COVID-19 pandemic among HCWs.

Around half of our participants reported perceived transgression by others (e.g., “I saw things that were morally wrong”) and perceived betrayal (“I feel betrayed by leaders whom I once trusted”). MIES scores were associated with the severity of clinical symptoms, including suicidal ideation, suggesting that HCWs face greater vulnerability in the COVID-19 era. These findings concord with previous studies demonstrating a correlation between moral injury and psychopathology among military veterans [13, 15, 16, 33–35]. Furthermore, a recent review examining traumatic responses among HCWs during the COVID-19 pandemic has highlighted the presence of trauma-related stress [36]. Our findings suggest that COVID-19 pandemic effects on HCWs resemble the lingering effects following a traumatic event. However, previous publications describing moral injury among HCWs during this pandemic have been purely commentary or theoretical, without adequately assessing moral injury levels or their implications [37–40]. This study is the first to assess moral injury levels and their relationships with a range of psychopathology among HCWs, providing an initial empirical basis for the strong relations between this phenotype and moral injury in HCWs.

We found disturbingly high levels of self-reported psychopathology. A relatively stable range of 52–62% of the surveyed HCWs reported probable anxiety across assessments, 54–58% probable depression, and 14–19% reported suicidal ideation on at least “several days” of the previous 2 weeks. Thirty-five percent reported probable COVID-19-related PTSD at baseline, decreasing to 24% by day 90. Other studies that surveyed psychopathology in HCWs found lower rates of anxiety (13–23%) and depression (18–23%) [4, 9, 41, 42]. One possible explanation may be the fluctuating but persisting and enduring course of the COVID-19 pandemic in the United States, generating greater stress, burnout, and moral injury that in turn increased anxiety and depression. An alternative explanation may relate to the absence of uniform COVID-19 policy in the United States during this time frame, which may have amplified psychopathology and moral injury. It is also possible that differences between HWC samples could account for the higher rates of psychopathology in our study. As moral injury has never been surveyed in the general population, this study provided an opportunity to explore moral injury in a civilian, albeit highly stressed and traumatized, sample.

Our findings emphasize the need for early detection and treatment of mental health difficulties among healthcare workers. Unfortunately, healthcare workers are often reluctant to seek mental health care, which increases the need for an intervention to facilitate their treatment-seeking intentions. Among barriers to care, stigma toward treatment is a profound obstacle, as some may perceive receiving treatment as a weakness or a failure to meet social or one’s own expectations [43, 44]. Long after the pandemic eventually loosens its grip, the psychiatric effects on HCWs may well not subside, leaving HCWs vulnerable and in need of assistance [19, 45, 46]. Studies are needed to examine how to increase the likelihood of seeking care, which is essential during this COVID-19 crisis.

### Limitations

Our study has several limitations. First, findings are limited to Amazon Mechanical Turk participants, who may not fully represent the HCW population, as they include mostly nurses (68%) and women (74%), compared to 30% registered nurses and 73% women in the healthcare occupation. Furthermore, 73% of participants described themselves as White, 13% as African American, and 11% as Asian, and 10% reported Hispanic ethnicity. These percentages are slightly divergent from the overall US population 2020 Census report of 72% White, 21% African American, 5% Asian, and 10% Hispanic. Second, clinical assessments, based on self-report questionnaires rather than formal diagnostic interview, were subject to over- or under-reporting [47]. Third, while the COVID-19 virus widely struck the US, our data lacked specific information on exposure to COVID-19 phenomena (e.g., exposure to death). Fourth, we lacked statistical power to compare HCW subgroups. Lastly, although moral injury appears not to be an exclusively military-related contrast, future studies need to establish the incremental validity of morally injurious outcomes, relative to symptoms of PTSD.

## Conclusions

More than 60% of HCWs surveyed in this study consistently reported symptoms of probable generalized anxiety, depression, and/or PTSD over the course of 3 months in late 2020. These outcomes were associated with moral injury and exposure to COVID-19. The overwhelming and unprecedented nature of the COVID-19 pandemic underscores the need for interventions aiming to reduce mental health stigma and increase treatment-seeking among HCWs [26, 48]. Employers and administrators should support and proactively encourage employees to access care when needed.

## Data Availability

The data that support the findings of this study are available on request from the corresponding author. The data are not publicly available due to privacy or ethical restrictions.
